# Development and Validation of an Algorithm to Accurately Identify Atopic Eczema Patients in Primary Care Electronic Health Records from the UK

**DOI:** 10.1016/j.jid.2017.03.029

**Published:** 2017-08

**Authors:** Katrina Abuabara, Alexa M. Magyari, Ole Hoffstad, Zarif K. Jabbar-Lopez, Liam Smeeth, Hywel C. Williams, Joel M. Gelfand, David J. Margolis, Sinead M. Langan

**Affiliations:** 1 Program for Clinical Research, Department of Dermatology, University of California San Francisco, San Francisco, California, USA; 2 Department of Health Policy & Management, University of California Berkeley School of Public Health, Berkeley, California, USA; 3 Department of Biostatistics, Epidemiology, and Bioinformatics, University of Pennsylvania Perelman School of Medicine, Philadelphia, Pennsylvania, USA; 4 Unit for Population-Based Dermatology Research, St John's Institute of Dermatology, King's College London, London, UK; 5 Faculty of Epidemiology & Population Health, London School of Hygiene and Tropical Medicine, London, UK; 6 Centre of Evidence-Based Dermatology, University of Nottingham, Nottingham, UK; 7 Department of Dermatology, University of Pennsylvania Perelman School of Medicine, Philadelphia, Pennsylvania, USA

**Keywords:** AE, atopic eczema, CI, confidence interval, PPV, positive predictive value, THIN, The Health Improvement Network

## Abstract

Electronic health records hold great promise for clinical and epidemiologic research. Undertaking atopic eczema (AE) research using such data is challenging because of its episodic and heterogeneous nature. We sought to develop and validate a diagnostic algorithm that identifies AE cases based on codes used for electronic records used in the UK Health Improvement Network. We found that at least one of five diagnosis codes plus two treatment codes for any skin-directed therapy were likely to accurately identify patients with AE. To validate this algorithm, a questionnaire was sent to the physicians of 200 randomly selected children and adults. The primary outcome, positive predictive value for a physician-confirmed diagnosis of AE, was 86% (95% confidence interval = 80–91). Additional criteria increased the PPV up to 95% but would miss up to 89% of individuals with physician-confirmed AE. The first and last entered diagnosis codes for individuals showed good agreement with the physician-confirmed age at onset and last disease activity; the mean difference was 0.8 years (95% confidence interval = –0.3 to 1.9) and –1.3 years (95% confidence interval = –2.5 to –0.1), respectively. A combination of diagnostic and prescription codes can be used to reliably estimate the diagnosis and duration of AE from The Health Improvement Network primary care electronic health records in the UK.

## Introduction

Atopic eczema (AE, synonymous with *atopic dermatitis* and commonly referred to as *eczema*) is one of the 50 most burdensome diseases worldwide (Vos et al., 2012, Weidinger and Novak, 2016). Therefore, there is great interest in understanding its causes, natural history, and potential associations with comorbid conditions. However, most studies rely on highly selected specialty clinic populations, cross-sectional studies, or self-reported data and are prone to bias and limited generalizability (Asher et al., 1995, Deckert et al., 2014). Representative population-level data with validated diagnoses and longitudinal follow-up are needed.

Electronic health data from primary care practices in the UK present an opportunity to directly address many of the unanswered questions about long-term outcomes in AE in particular. They are representative of the general population, include relatively long-term follow-up of both children and adults, and are appropriate for the study of AE because 97% of patients are managed by general practitioners in the UK (Emerson et al., 1998, Schofield et al., 2009). However, these data were created for administrative and clinical purposes, not designed specifically for research, and it is therefore critically important that the validity of AE diagnoses in these data sources is understood (Manuel et al., 2010). Because AE is a heterogeneous and episodic condition with nonspecific terminology, there exists high potential for misclassification of diagnosis and duration of disease. There is no single diagnostic test for AE, and it can be challenging to diagnose in population-based studies because of its variability in morphology, distribution, and periodicity. The diagnosis relies on clinical judgment based on a combination of history and physical examination. Previous studies using UK primary care data to identify patients with AE report wide variations in prevalence from 0–38% based on the coding algorithm used (Anandan et al., 2009, Carey et al., 2003, McKeever et al., 2001, McKeever et al., 2002, McKeever et al., 2004, Punekar and Sheikh, 2009, Simpson et al., 2002, Simpson et al., 2009). Moreover, there is some evidence that chronic diseases, such as AE, may be more poorly recorded over time in UK general practice data, because general practitioners are not required to enter codes on each occasion for chronic conditions (Jordan et al., 2004, Khan et al., 2010).

This study aimed to enhance identification of patients with AE within electronic health records. The objectives were to develop and validate a diagnostic algorithm for AE that identifies cases based on codes and, secondarily, to examine the agreement between physician report and codes for AE disease onset, duration, and severity.

## Results

### Algorithm development

A list of potential AE diagnosis and treatment codes was developed by using a keyword search and examining affiliated codes (see Supplementary Table S1 online), and the five most common and specific codes for AE were chosen to identify those likely to have AE: M111.00 atopic dermatitis/eczema, M1120.0 infantile eczema, M113.00 flexural eczema, M11400 allergic/intrinsic eczema, and M12z100 eczema not otherwise specified. When we examined the frequency of medical codes among individual patients, we found that including 32 codes likely to be related to AE rather than only the five most common codes only slightly increased the number of individuals identified but that including up to 74 possible AE codes nearly doubled the number of individuals identified (Table 1). The distribution of some codes varied between children and adults; for example, M1120.0/infantile eczema was more commonly used in children.

**Table 1:** Distribution of codes in the entire THIN database, %

Codes	Total	Children (Ages 0–17)^1^	Adults (Ages 18+)^1^
	N = 9,775,618	n = 1,404,158	n = 8,371,460
Diagnosis codes			
AD/eczema M111.00	6	13	5
Infantile eczema M112.00	1	7	0
Flexural eczema M113.00	1	2	0
Allergic/intrinsic eczema M114.00	0	0	0
Eczema not otherwise specified M12z100	6	8	6
One or more of the five codes listed above	13	23	11
Two or more of the five codes listed above	4	10	4
One or more of 32 likely eczema codes^2^	14	25	13
One or more of 74 possible eczema codes^2^	29	47	26
Prescription codes			
One or more prescription for any AD-related therapy^3^	45	57	42
One or more prescription for a topical steroid or calcineurin inhibitor^3^	39	42	38
One or more prescription for an AD-related systemic treatment^3^	1	0	1
Other codes			
One or more exclusionary condition^4^	7	3	8
One or more dermatology consult code	4	2	5
One or more biopsy or patch testing code	1	0	1

Abbreviation: AD, atopic dermatitis.

1 Ages as of January 2013; among adults, codes may have occurred before age 18 years.

2 See Supplementary Table S1.

3 Includes topical skin preparations, topical steroids, topical calcineurin inhibitors, topical anti-infective treatments, and systemic treatments (including methotrexate, azathioprine, mycophenolate, cyclosporine, biologics, or phototherapy); see Supplementary Table S2.

4 See Supplementary Table S3 online.

Despite the chronicity of AE, any of the five most common diagnosis codes were rarely repeated in the database; overall, patients had a mean of 1.2 (standard deviation = 0.5) codes during 5.6 years (standard deviation = 8.0) of follow-up. Because AE is by definition a chronic condition, it was important to include more than one code in our algorithm, but requiring individuals to have two or more diagnosis codes would exclude more than 80% of the potential AE population. Therefore, the distribution of treatment codes was also examined. In the UK, medical record codes and treatment codes can be entered independently (i.e., a prescription code does not require an associated diagnostic code). Prescriptions, including emollient preparations, are available through the National Health Service, so we examined prescription codes for all potential relevant therapies including topical emollients, topical steroids, topical calcineurin inhibitors, topical anti-infective treatments, and systemic immunomodulatory medications (including methotrexate, azathioprine, mycophenolate, cyclosporine, or biologics) based on British National Formulary groupings and phototherapy codes (Joint Formulary Committee, 2015) (see Supplementary Table S2 online). Because prescriptions are free of charge for children only, we stratified our analyses by age (i.e., children younger than 18 years vs. adults). We also specifically examined the use of topical steroids and topical calcineurin inhibitors (which are likely to be more specific for AE). To ensure that we captured patients with chronic AE in our algorithm, we chose to include patients with at least one of the five medical codes frequently used for AE as listed *and* at least two treatment codes for any AE-related therapy on separate dates (at any time point relative to the AE diagnosis, because symptoms may precede the actual diagnosis).

### Physician survey

**Figure 1:**
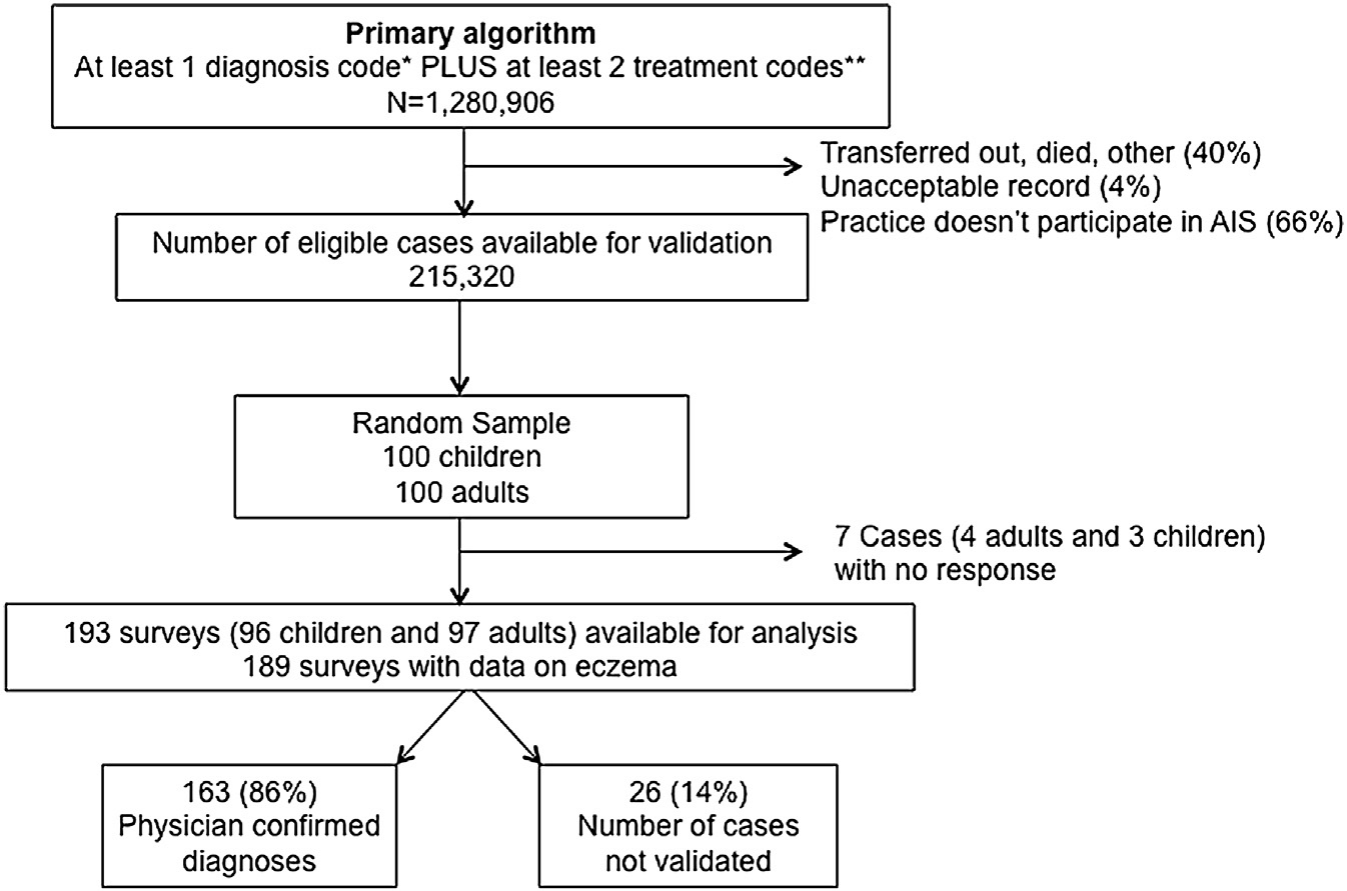
**Flow chart showing the sampling of patients from THIN and resulting classification.**^∗^Any of the five most commonly used Read codes (atopic dermatitis/eczema M111.00, infantile eczema M112.00, flexural eczema M113.00, allergic/intrinsic eczema M114.00, or eczema not otherwise specified M12z100). ^∗∗^Any code for topical emollients, topical steroids, topical calcineurin inhibitors, topical anti-infective treatments, systemic immunomodulatory medications (including methotrexate, azathioprine, mycophenolate, cyclosporine, or biologics) or phototherapy on separate days; see Supplementary Table S2). AIS, Additional Information Services; THIN, The Health Improvement Network.

To validate the algorithm for AE, we surveyed the physicians of a random sample of 100 children (<18 years of age) and 100 adults (Figure 1). The response rate was 97% overall (96% for adults and 97% for children), and there was no significant difference in response rate by age or sex. The algorithm for identifying patients with AE performed well, and there were no significant differences in codes between those with and without physician-confirmed AE (Table 2). The positive predictive value (PPV) for a single diagnostic code and at least two treatment codes was 86% overall (95% confidence interval [CI] = 80–91) and was higher among children (90%) than adults (82%), although this difference was not statistically significant (Pearson χ^2^ = 2.76, *P* = 0.097).

**Table 2:** Survey sample characteristics

Survey Responses	Total^1^	Confirmed Eczema^1^	No Eczema^1^	Chi-Square or Fisher’s *P*-Value
All participants, n (%)	200 (100)	163 (81.5)	26 (13)	
Diagnosis codes, n (%)				
AD/eczema M111.00	116 (58)	98 (60)	13 (50)	0.330
Infantile eczema M112.00	30 (15)	24 (15)	4 (15)	0.930
Flexural eczema M113.00	16 (8)	13 (8)	2 (8)	0.960
Allergic/intrinsic eczema M114.00	3 (2)	3 (2)	0 (0)	0.486
Eczema not otherwise specified M12z100	86 (43)	73 (45)	9 (34)	0.331
Mean number (SD) of the five eczema codes listed above	1.3 (0.5)	1.3 (0.6)	1.1 (0.3)	0.051
Mean number (SD) of 32 likely eczema codes^2^	2.6 (2.9)	2.8 (3.1)	1.7 (1.2)	0.070
Mean number (SD) of 74 possible eczema codes^2^	4.0 (3.8)	4.1 (3.9)	3.2 (3.3)	0.271
Prescription codes				
Mean number (SD) of prescriptions for any AD-related therapy^3^	16.3 (24.5)	17.5 (26)	11.1 (15.0)	0.226
Mean number (SD) of topical steroid or calcineurin inhibitor prescriptions^3^	9 (15)	6.6 (8.9)	6.5 (7.9)	0.953
Mean number (SD) of AD-related systemic treatment codes^3^	0.4 (5.8)	0.5 (6.4)	0 (0)	0.691
Other				
Mean number (SD) of exclusionary diagnostic codes^4^	0.4 (1.7)	0.3 (0.7)	0.5 (1.6)	0.281
Total (%) with at least one exclusionary condition^4^	29 (15)	25 (15.3)	3 (11.5)	
Mean number (SD) of diagnostic procedure (biopsy or patch testing) codes^4^	0	0	0	N/A
Mean number (SD) of dermatology consultation codes^4^	0.2 (1.1)	0.3 (1.6)	0.0 (0.2)	0.308
Total (%) with at least one dermatology consultation code	19 (10)	18 (11)	1 (4)	
History of atopy, n (%)^5^	64 (39)	56 (41)	6 (24)	0.110
Male, n (%)	100 (50)	86 (53)	9 (35)	0.086

Abbreviation: AD, atopic dermatitis; N/A, not applicable; SD, standard deviation.

1 Columns do not sum to 200 because of missing values (seven unreturned surveys and four returned surveys missing a response to the eczema question).

2 See Supplementary Table S1.

3 Includes topical skin preparations, topical steroids, topical calcineurin inhibitors, topical anti-infective treatments, and AD-related systemic treatments (including methotrexate, azathioprine, mycophenolate, cyclosporine, biologics, or phototherapy); see Supplementary Table S2.

4 See Supplementary Table S3.

5 Per physician response on survey; defined as a history of *other atopic disease (e.g., asthma or allergic rhinitis) for adults or a family history of atopic disease in a first degree relative if aged under 4 years*.

When we examined whether the use of more stringent criteria would improve the prediction of physician-confirmed AE, we found that adding additional criteria to the algorithm had the potential to increase the PPV but would result in smaller numbers of individuals being detected (Table 3). For example, requiring two AE codes would increase the PPV to 91% but would detect only 83 of 163 (51%) of those with physician-confirmed AE. Similarly, requiring a dermatology consultation code in addition to the AE and prescription codes would increase the PPV to 95% but would detect only 18 of 163 (11%) of those with physician-confirmed AE. Requiring the prescriptions to be for medications more specific to AE (i.e., topical steroids or calcineurin inhibitors) did not significantly change the PPV.

**Table 3:** Positive predictive value of coding algorithms

	True Positive/All Positive	% of Patients with Confirmed Eczema Identified	All		Children (Ages 0–17)		Adults (Ages 18+)	
			PPV, %	95% CI	PPV, %	95% CI	PPV, %	95% CI
Baseline algorithm; one of five eczema codes + at least two treatment codes on separate dates (survey selection criteria)	163/189	N/A	86	80–91	90	83–96	82	73–89
Alternative algorithms^1^								
Baseline algorithm; at least one treatment is a topical steroid/TCI code	157/183	96	86	80–91	90	81–95	82	73–89
Baseline algorithm; at least two treatments are topical steroid/TCI codes	133/153	82	87	81–92	91	82–97	84	74–91
Baseline algorithm; at least one treatment is a topical steroid/TCI code either 3 months before or up to 1 year after the eczema code	81/92	50	88	80–94	92	80–98	84	70–94
Baseline algorithm + an additional eczema code (two eczema codes total)	83/91	51	91	83–96	94	82–99	88	74–96
Baseline algorithm + an additional eczema code (two eczema codes total); at least one treatment is a topical steroid/TCI code	82/90	50	91	83–96	94	83–99	88	74–96
Baseline algorithm + an additional eczema code (two eczema codes total); at least two treatments are topical steroid/TCI code	133/153	82	87	81–92	91	82–97	84	74–91
Baseline algorithm + no exclusionary condition code	138/161	85	86	79–91	89	81–95	82	71–90
Baseline algorithm + asthma or rhinitis code	52/56	32	93	83–98	95	76–100	91	77–98
Baseline algorithm + dermatology consult code	18/19	11	95	74–100	100	54–100	92	64–100

Abbreviations: CI, confidence interval; PPV, positive predictive value; TCI, topical calcineurin inhibitor.

1 See Supplementary Tables S2 and S3 for lists of codes.

The average age of onset and oldest age of disease activity requiring physician contact estimated using codes from the database were similar to what physicians reported (Table 4). The mean estimated ages at onset using the first diagnosis code or the first treatment code were both slightly younger than the physician estimate (mean difference = 0.8 years, 95% CI = –0.3 to 1.9 and mean difference = 0.4 years, 95% CI = –0.8 to 1.7, respectively), and 76% of estimates were within 1 year of each other. The mean estimated age at last date of AE activity using the last diagnosis code or last treatment code were both older than the physician estimate (mean difference = –1.3 years, 95% CI = –2.5 to –0.1 and mean difference = –3.9 years, 95% CI = –5.3 to –2.4, respectively), and 79% of estimates within 5 years of each other. Bland-Altman plots for all estimates are shown in Supplementary Figure S1 online. When we stratified these estimates by age comparing children under age 18 years versus adults we found similar results (see Supplementary Table S4).

**Table 4:** Age in years at diagnosis or at last disease activity requiring contact with the physician

	Distribution of Estimates by Source		Difference Between Physician Estimate from Survey and Database	
	Mean	95% CI	Mean	95% CI
Age at diagnosis (n = 160)				
Physician survey	17.9	14.3–21.4	N/A	N/A
Database				
First diagnosis code^1^	17.1	13.5–20.6	0.8	– 0.3 to 1.9
First prescription for any eczema treatment^2^	17.4	13.9–21.0	0.4	– 0.8 to 1.7
If no symptoms in the year before the last visit date, age at last disease activity (n = 53)				
Physician survey	20.7	14.3–27.2	N/A	N/A
Database				
Last diagnosis code^1^	22.0	15.6–28.5	–1.3	– 2.5 to –0.1
Last prescription for any eczema treatment^2^	24.6	14.3–27.2	–3.9	– 5.3 to –2.4

Abbreviations: CI, confidence interval; N/A, not applicable.

1 Any of the five most commonly used codes (atopic dermatitis/eczema M111.00, infantile eczema M112.00, flexural eczema M113.00, allergic/intrinsic eczema M114.00, or eczema not otherwise specified M12z100).

2 See Supplementary Table S2.

In our sample, 48 patients were reported by the physician to have had symptoms in the year prior to their last visit, 27 (56%) of whom were assessed as having mild disease and 19 (40%) of who were assessed as having moderate disease based on the severity descriptions in the National Institute for Health and Care Excellence guidelines. Patients with moderate disease had more treatment codes during that year than patients with mild disease (median = 5 vs. 2, *P*-value for two-sample Wilcoxon rank sum test = 0.887). None were reported to have severe disease, limiting our ability to draw any conclusions about the validity of medical record codes to predict disease severity.

Finally, we assessed whether physicians would be able to adequately respond to the UK Working Party criteria (originally designed for in-person assessment), enabling us to compare a set of well-validated criteria for use in large epidemiologic studies with our outcomes in routinely collected electronic health data. For each question, we gave physicians the option of choosing *Don’t know.* The high number of uncertain responses resulted in poor ability to discriminate between those with and without AE (see Supplementary Table S5 online). We found that only 52 (32%) of those with physician-confirmed AE in our sample met the criteria (an itchy skin condition plus at least three of the following: flexural involvement, history of asthma/hay fever, history of generalized dry skin, onset of rash at younger than age 2 years, and visible flexural dermatitis).

## Discussion

### Interpretation of main findings

Patients with AE were accurately identified if they had at least one AE diagnostic code and at least two prescription codes for AE-related treatments in a large electronic medical record database representative of the general population in the UK. The PPV, or probability that individuals identified by our algorithm truly have the disease as determined by their doctors, was 86%, which is similar to the PPV of coding algorithms for other chronic diseases in routinely collected data (Khan et al., 2010). The PPV was higher in children, but the algorithm still performed well to identify adults with AE.

This study indicates that the types, number, and frequency of codes used to identify AE patients in routinely collected data are important because small differences have the potential to cause substantial misclassification. After examining the distribution of all codes potentially related to AE, we chose to use the five most common AE codes in addition to treatment codes for the primary algorithm. As shown in Table 1, expanding the definition from five to 32 codes (likely related to AE but rarely used) would have increased the proportion of the population identified only from 13% to 14%, so we opted for the more parsimonious algorithm. In contrast, using a single code to define AE, for example AD/eczema (M111.00), would identify far fewer individuals (only 6% of the population). Although it was impractical and prohibitively costly to sample enough physicians to calculate the sensitivity, specificity, and predictive value of each of these variations, we present the proportion of patients identified by each set of codes to illustrate the potential magnitude of misclassification. We were able to calculate post hoc changes in the PPV caused by adding criteria to our algorithm. Inclusion of a second diagnosis code, allergy code, or consult code all increased the PPV but would have identified far fewer patients. The ideal balance between these factors depends on the research question. For example, an algorithm with a very high PPV that captures only a fraction of those with disease may be acceptable for a case-control study. On the other hand, the ideal algorithm for a prevalence study would aim to assess the total population burden accurately and may include more patients with mild or marginal disease.

Because AE is a chronic condition, we explored the possibility of using codes from more than one time point to identify patients. In the UK, providers are not required to re-enter codes for chronic conditions, and only 36% of individuals had more than one AE diagnosis code. Treatment codes, which can be entered independently from diagnostic codes, were used more frequently and were therefore included in the algorithm. When selecting the treatments, we opted for an inclusive approach and used all potential AE-related treatments, even emollients, as listed under British National Formulary categories. This approach may include treatments not specifically for AE, so we examined the performance of a more limited definition of treatments (only topical steroids or topical calcineurin inhibitors) and found that it did not change the PPV but would identify 4–18% fewer patients (Table 3). Of note, 22% of individuals with one of the five most common medical codes never received any treatment codes. Our algorithm excluded these patients, some of whom may have had mild untreated disease.

Because we randomly selected individuals with AE diagnoses at any time point, only a fraction had disease activity during the year before their last visit, resulting in too few numbers to meaningfully assess the validity of codes relative to disease severity. Additional research is necessary to validate whether codes can be used to ascertain severity and disease flares in routinely collected data.

### Comparability to other studies

Three other studies attempted to validate routinely collected data for identifying individuals with AE. Two examined the use of medications alone and found they had poor discriminatory power to identify patients with AE in The Netherlands and Sweden (Mulder et al., 2016, Ortqvist et al., 2013). The distribution of treatment codes in our data, as shown in Table 1, also suggested that the were not likely to selectively identify patients with AD, which is why we designed our algorithm to incorporate both diagnosis and treatment codes as described above. The third study compared International Classification of Diseases, Ninth Revision, codes from a tertiary care population in the US with Hanifin and Rajka and UK Working Party criteria found in the medical record and found poor overlap (Hsu et al., 2016), possibly because of the lack of standardized recording of specific diagnostic features in the medical record. We assessed whether it was possible to compare our results to the UK Working Party diagnostic criteria, which have been used for epidemiological studies in multiple international settings but were developed for in-person assessment (Brenninkmeijer et al., 2008, Williams et al., 1994). Because physicians responded *Don’t know* to so many of the UK Working Party questions in our survey, we were unable to make meaningful comparisons. We hypothesize that the high rates of uncertainty were because there were not enough data in the medical record to enable physicians to answer all of the required questions and therefore caution against using these as a criterion standard from medical record review when they were not systematically assessed. It is also possible that those deemed to have AE by their physicians simply would not fulfill the criteria if they had been ascertained fully, and further specially designed studies are needed to test this notion.

### Strengths and weaknesses

Strengths of our study include the use of diagnosis and treatment codes, stratified sampling among children and adults, a large representative database with longitudinal follow-up, and physician confirmation of disease as the criterion standard. We sampled general practice physicians rather than dermatologists because 97% of patients with AE are managed by general practitioners in the UK, and sampling specialists would have limited the generalizability of the results (Emerson et al., 1998, Schofield et al., 2009).

Ideally, patients would have been assessed in person to confirm their diagnoses. Because this was not possible through the Additional Information Services in THIN, we queried their physicians instead. The physicians were asked to assess the patient based on their recall and review of the medical record. This approach was chosen over a medical record review because it allowed for direct assessment as to whether the physician really believed the patient had AE (regardless of coding).

Our results are directly generalizable only to THIN, although the algorithm is likely to perform similarly in the other UK primary care databases that have substantial overlap (the Clinical Practice Research Datalink [https://www.cprd.com/] and other UK primary care data sources including QResearch [http://www.qresearch.org/]). Validation studies are inherently context specific, and the PPV of our algorithm may vary in settings where the prevalence of AE and data structure differ. For example, we found that adding a dermatology consultation code to our baseline algorithm increased the PPV to 90% (95% CI = 74–100) (Table 3); however, it identified only 11% of the patients with confirmed eczema because very few patients are referred to specialists in the UK. In the US, where the proportion of patients who are referred to a specialist is higher (it is estimated 43% of pediatric AE visits were to generalists between 1997 and 2004) (Horii et al., 2007), adding a dermatology consultation code to the baseline algorithm is likely to identify a higher proportion of patients with confirmed AE. If our algorithm were used in settings where patients do not receive prescriptions for emollients or other topical preparations or anti-infective treatments, its performance may be more comparable to the first two alternative algorithms listed in Table 3, which are based on the use of topical steroids and calcineurin inhibitors alone. We emphasize the importance of carefully examining the distribution and types of codes before undertaking a study using electronic medical record data, and we present the distribution of categories of codes in Table 1 so that researchers can evaluate how applicable our results may be to their data.

### Implications for future research

Validation studies that ensure patients are accurately identified are a high priority, to enable the use of increasingly available and robust sources of routinely collected electronic health data (De Coster et al., 2006), but they have not been widely used in the AE literature to date. This study showed that AE patients can be accurately identified in the UK THIN database and that changes in the number, type, or frequency of codes used could result in large differences in the number of patients identified. Additional work is necessary to determine the PPV of our algorithm in other contexts. We highlight factors to consider when examining the frequency and distribution of diagnostic and treatment codes in any electronic medical record database, which are important for researchers to avoid misclassification bias. Efforts are underway to determine how AE patients have been identified in published studies using electronic health data (Dizon et al., 2016), and we encourage the research community to work toward developing standards for methodology and reporting to improve comparability of studies and advance our understanding of AE.

## Methods

### Study design

Our study consisted of two parts: a longitudinal cohort study to develop a diagnostic algorithm and a physician survey to validate it. We followed guidelines for reporting of validation studies and reporting of studies conducted using observational routinely collected health data (Benchimol et al., 2011, Benchimol et al., 2016).

### Participants/data source

THIN is a database comprising the electronic health records of people registered with participating general practices. THIN is broadly representative of the general UK population in terms of age, sex, ethnicity, and geography and is one of three major UK primary care databases (Shephard et al., 2011). We chose this data source because it is one of the world’s largest sources of anonymized longitudinal data from primary care practices, with over 85 million patient-years of follow-up, and because we had institutional access and experience using the data (Margolis et al., 2007, Margolis et al., 2008, Ogdie et al., 2015, Seminara et al., 2011). Previous validation studies have shown that the recording is highly accurate and nearly complete, and THIN has been used to study multiple chronic conditions. Participating practices are remunerated for recording data on clinical diagnoses, test results, prescriptions, and referral data via the Read/ Oxford Medical Information System coding framework, which is based on the International Classification of Diseases coding system. The raw data are updated monthly and undergo extensive quality control and validity checks by a centralized research team before release. Practices may choose to participate in the Additional Information Services Program, which administers surveys to consenting physician practices. Approximately 60% of all THIN practices actively participated in this program when our survey was administered in October 2015.

### Algorithm development

A list of potential AE diagnosis and treatment codes were developed by using a keyword search and examining affiliated codes (see Supplementary Table S1). The distribution of codes was examined, and in consultation with a panel of experts on AE epidemiology and use of routinely collected data (HCW, DM, LS, SML, and KA) a parsimonious algorithm was developed to identify patients most likely to have AE.

### Physician survey

The survey was sent to the physicians of a random sample of 100 children (<18 years of age) and 100 adults with acceptable records who were alive and currently enrolled in practices participating in the Additional Information Services (Figure 1). The primary outcome was the PPV, or probability that subjects identified by the algorithm truly have the disease, because this measure is the most relevant for avoiding misclassification bias in subsequent studies of AE (Choi, 1992) (Supplementary Figure S2). Assuming a physician response rate of 90% (based on prior studies using physician confirmation of chronic disease in routinely collected data [Khan et al., 2010, Seminara et al., 2011]), a sample of 200 patients should have enabled us to obtain a 95% CI of 0.85–0.94 around an a priori estimated PPV of 0.90. Given funding constraints, we chose to sample only patients with codes suggestive of AE. Sampling additional subjects without AE codes would have enabled us to also calculate sensitivity and specificity of the algorithm.

A standardized letter was sent to each practice requesting completion of a 1-page survey (see Supplementary Figures S3 and S4 online), and physicians received monthly reminders for completion and compensation for their time. If the diagnosis of AE was confirmed, we then asked the physician to (i) provide a global assessment of average AE severity over the past 12 months, (ii) confirm the age at AE onset, and (iii) confirm whether the patient still has active AE or whether the patient’s AE is in remission. Although many eczema-specific severity scales have been developed and validated for assessment of patient outcomes in clinical trials, few are designed to address long-term severity (Schmitt et al., 2007). Therefore, to assess severity, we used descriptions of mild, moderate, and severe disease from the UK National Institute for Health and Care Excellence guidelines for management of eczema (National Institute for Health and Care Excellence, 2007). Finally, to determine whether our results could be compared with another widely used definition of AE in large epidemiologic studies, the survey included the UK Working Party refinement of Hanifin and Rajka’s diagnostic criteria questions (Brenninkmeijer et al., 2008, Williams et al., 1994).

AE is a clinical diagnosis, and biopsy and laboratory tests are nonspecific; therefore, we relied on the physician’s confirmation of the diagnosis as the criterion standard. This approach is consistent with other validation studies of chronic conditions in medical record databases in UK primary care databases (Ogdie et al., 2014, Seminara et al., 2011, Soriano et al., 2001). Physicians were asked to fill out the survey based on their knowledge of the patient and review of his or her medical record.

### Analysis

For the 200 patients whose physicians were surveyed, differences in codes between those with and without physician-confirmed AE were examined, and the PPV of our algorithm for identifying AE patients was calculated. The PPVs of alternative algorithms with additional criteria for identifying patients with AE were also calculated. Next, the age of disease onset and “remission” reported in the physician survey were compared with dates calculated from the database using the first and last AE diagnosis and prescription codes. Agreement was assessed descriptively using Bland-Altman plots (Bland and Altman, 1986). All analyses were stratified by age (i.e., children younger than 18 years vs. adults). Analyses were performed using Stata, version 14 (Stata Corporation, College Station, TX).

### Ethics

Approval was obtained from the Scientific Research Council of THIN and the University of Pennsylvania institutional review board.

## ORCIDs

Zarif K Jabbar-Lopez: http://orcid.org/0000-0003-4127-8263

Hywel Williams: http://orcid.org/0000-0002-5646-3093

Katrina Abuabara: http://orcid.org/0000-0002-7736-6946

## Conflict of Interest

JMG served as a consultant and received grants from Sanofi and Regeneron. LS and SML are supported by the Wellcome Trust. KA is supported by the National Institutes of Health, the Dermatology Foundation, and the Robert Wood Johnson Foundation. The other authors state no conflict of interest.
