# Supplementary material for: Development and Validation of an Algorithm to Accurately Identify Atopic Eczema Patients in Primary Care Electronic Health Records from the UK
Source: J Invest Dermatol. 2017 Aug;137(8):1655–62. doi: 10.1016/j.jid.2017.03.029 (PMC5883318; doi:10.1016/j.jid.2017.03.029)
Supplement: Supplementary Tables S1–S5 and Supplementary Figures S1–S4 [file mmc1.pdf]

SUPPLEMENTARY TABLES AND FIGURES

Supplementary Table S1. Results of keyword* search and frequency of potential eczema-related codes (*eczema, dermatitis, atopic, rash, itch, dermatology, dermatologist, and biopsy with skin, punch, and/or shave)

READ Code	Description	Frequency	Possibly Related	Likely Related
1D14.00	C/O: a rash	1,728,186	1	0
M12z100	Eczema NOS	1,204,114	1	1
M111.00	Atopic dermatitis/eczema	1,055,500	1	1
9N1S.00	Seen in dermatology clinic	1053407	0	
R021z00	[D]Rash and other nonspecific skin eruption NOS	777621	1	0
M12z000	Dermatitis NOS	347513	1	0
R021.00	[D]Rash and other nonspecific skin eruption	244152	1	
M101.00	Seborrhoeic dermatitis	195401	0	0
M112.00	Infantile eczema	168677	1	1
M110.13	Nappy rash	159475	1	0
G831.11	Varicose eczema	155,678	0	0
M12..11	Contact dermatitis	148207	0	0
1D15.00	C/O: itching	144182	1	
14F1.00	H/O: eczema	138851	1	1
2227.00	O/E - rash present	137446	1	
2227.11	O/E - allergic rash	130257	1	0
M18z.12	Itch	120299	1	
M12z200	Infected eczema	114949	1	1
A57z.11	Non specific viral rash	105735	0	
M101.12	Seborrhoeic eczema	95479	1	1
2227.12	O/E - itchy rash	87639	1	0
2I14.00	O/E - a rash	75499	1	
1B86.11	Itchy eye symptom	71211	0	
M113.00	Flexural eczema	70016	1	1
1D15.11	Scalp itchy	54380	0	
ZL5AB00	Referral to dermatologist	53953	0	
M1...11	Dermatitis/dermatoses	48786	1	0
M101.11	Seborrhoeic dermatitis capitis	44788	0	0
222D.00	O/E - Rash absent	36496	0	
AB23012	Nappy rash - candidal	36063	0	
M153500	Perioral dermatitis	35060	1	0
ZL9AC00	Seen by dermatologist	31253	0	
M110.00	Napkin dermatitis	30774	1	0
M12z300	Hand eczema	27945	1	1

READ Code	Description	Frequency	Possibly Related	Likely Related
M12..00	Contact dermatitis and other eczemas	23759	0	0
M07z.14	Infected dermatitis	22130	1	0
M180.11	Perianal itch	19279	0	
M114.00	Allergic (intrinsic) eczema	18659	1	1
7G05B00	Excision biopsy of skin lesion	18092	0	
M12..12	Contact eczema	18064	0	0
G831.00	Varicose veins of the leg with eczema	17843	0	0
2FW0.00	O/E - scalp rash	17133	0	
M12z111	Discoid eczema	16608	1	1
H330.00	Extrinsic (atopic) asthma	16348	0	
M251.11	Heat rash	14800	0	
1B86.00	Has an itchy eye	14057	0	
M102.00	Infectious eczematoid dermatitis	12982	1	0
7G0A.00	Punch biopsy of skin	12205	1	
8HVI.00	Private referral to dermatologist	11761	0	
7G0Cz00	Other biopsy of skin NOS	11128	1	
M128.00	Allergic contact dermatitis	10905	0	0
9NJ2.00	In-house dermatology	10236	0	
F500300	Chondrodermatitis nodularis helicis	9534	0	0
12H1.00	FH: Eczema	9435	0	0
M251.13	Sweat rash	8584	0	
7G0C.00	Other biopsy of skin	8409	1	
M11..00	Atopic dermatitis and related conditions	6732	1	1
2FU..00	O/E - erythematous rash	6658	1	0
M130.11	Drug induced rash	6342	0	
M110000	Candidal nappy rash	6292	0	
M12zz00	Contact dermatitis NOS	6201	0	0
9b9D.00	Dermatology	6049	0	
M11z.00	Atopic dermatitis NOS	6016	1	1
8H4S.00	Referral to dermatology special interest GP	5971	0	
M12z.00	Contact dermatitis NOS	5849	0	0
M102.11	Pustular eczema	5474	1	1
M127300	Photodermatitis	5444	0	
F502411	Eczema of external ear	4993	1	1
9NJB.00	In-house dermatology follow-up appointment	4916	0	
M184.00	Dermatitis artefacta	4471	0	
9NJA.00	In-house dermatology first appointment	4448	0	
F4D3112	Contact eczema - eyelids	4114	0	0
ZLD3C00	Discharge by dermatologist	3974	0	
26C4.00	Nipple eczema	3596	1	1

READ Code	Description	Frequency	Possibly Related	Likely Related
2F...11	O/E - dermatology exam.	3580	0	
F4C0600	Acute atopic conjunctivitis	3487	0	
7E02300	Punch biopsy of cervix uteri	3410	0	
2FO..00	O/E - discoid rash	3322	1	0
M140.00	Dermatitis herpetiformis	3234	0	
M183100	Neurodermatitis circumscripta	3086	1	
M2y4211	Vesicular rash	3019	0	
Myu2200	[X]Exacerbation of eczema	2834	1	1
M110.12	Diaper rash	2776	0	
7G0B.00	Shave biopsy of skin	2730	1	
M12y800	Contact dermatitis due to metals	2617	0	
M127.00	Contact dermatitis due to solar radiation	2526	0	
F4D3000	Eczematous eyelid dermatitis	2477	1	1
F4D3000	Eczematous eyelid dermatitis	2477	1	1
F4D3100	Contact or allergic eyelid dermatitis	2462	0	
F502400	Acute eczematoid otitis extern	2343	1	1
M131.00	Ingestion dermatitis due to food	2341	0	
M116.00	Neurodermatitis - diffuse	2289	1	
F4D3111	Allergic dermatitis - eyelid	2257	1	
M1y0.00	Nummular dermatitis	2238	1	
M1B..11	Juvenile plantar dermatitis	2217	1	
1N01.00	No itch	2140	0	
M120.00	Contact dermatitis due to detergents	2035	0	
7G0C300	Incision biopsy of skin	2011	0	
M118.00	Infantile seborrhoeic dermatitis	2001	0	
AB03.11	Dhobie itch	1976	1	
ZL18A00	Under care of dermatologist	1938	0	
M127000	Unspecified contact dermatitis due to solar radiation	1552	0	
A540.00	Eczema herpeticum - Kaposi's varicelliform eruption	1492	1	1
9NlB.00	Seen by GP with special interest in dermatology	1399	0	
M07y.11	Pustular eczema	1374	1	1
M124111	Elastoplast contact dermatitis	1351	0	
M124.00	Contact dermatitis due to other chemical products	1329	0	
M130.00	Ingestion dermatitis due to drugs	1302	0	
M070.11	Purulent dermatitis	1205	0	
ZLE6B00	Discharge from dermatology service	1190	0	
M12z400	Erythrodermic eczema	1154	1	1
M28y.11	Nettle rash	1134	0	
2I1B.00	Blanching rash	1119	0	
2I1C.00	Non-blanching rash	1088	0	

READ Code	Description	Frequency	Possibly Related	Likely Related
M124100	Contact dermatitis due to adhesive plaster	1028	0	
7G05A00	Excision biopsy of lesion of skin of head or neck	999	0	
9NI0.00	Dermatology outreach clinic	996	0	
2FU0.00	O/E - dribble rash	996	1	
M117.00	Neurodermatitis - atopic	989	1	1
M123800	Contact dermatitis due to scabicides	982	0	
1N04.00	Itching of skin lesion	954	1	
8H2D.00	Admit dermatology emergency	925	0	
M244111	Barbers' rash	923	0	
M123z00	Contact dermatitis due to medicament NOS	839	0	
2FR..00	Butterfly rash	835	0	
2536.00	O/E - mouth rash	805	1	
M12..13	Occupational dermatitis	786	0	
A512.00	Contagious pustular dermatitis	676	0	
M153511	Circumoral dermatitis	635	1	
F500211	Chondrodermatitis nodularis helicis	629	0	
M110.11	Ammonia dermatitis	625	0	
7G0A100	Punch biopsy of lesion of skin NEC	517	1	
G832.00	Varicose veins of the leg with ulcer and eczema	507	0	0
M126.00	Contact dermatitis due to plants	496	0	
M12y000	Contact dermatitis due to cosmetics	461	0	
8HHX.00	Referral to dermatology nurse specialist	450	0	
M118000	Infantile seborrhoeic dermatitis capitis	420	0	
M121.00	Contact dermatitis due to oils and greases	408	0	
R021100	[D]Rash on genitals	401	0	
Myu2.00	[X]Dermatitis and eczema	388	1	1
7G0Az00	Punch biopsy of skin NOS	369	1	
M11A.00	Asteatotic eczema	366	0	0
M123.00	Contact dermatitis due to drugs and medicaments	364	0	
7G0C000	Biopsy of lesion of skin of head or neck NEC	361	1	
M153600	Periocular dermatitis	357	1	
8HM3.00	Listed for Dermatology admissn	335	0	
8H3I.00	Non-urgent dermatology admisn.	324	0	
AB23011	Monilial nappy rash	313	0	
A54x300	Herpesviral vesicular dermatitis	295	0	
M2y4811	Juvenile plantar dermatitis	289	0	
M122.00	Contact dermatitis due to solvents	247	0	
9Ni6.00	DNA dermatology special interest general practitioner clinic	247	0	
8Hkq.00	Referral to community dermatology service	245	0	

READ Code	Description	Frequency	Possibly Related	Likely Related
M1y2.00	Gravitational eczema	231	0	0
2FT4.00	Itching of pigmented skin lesion	226	1	
M129200	Irritant contact dermatitis due to other chemical products	211	0	
9NJC.00	In-house dermatology discharged from care	210	0	
M115.00	Besnier's prurigo	208	1	1
M13..00	Ingestion dermatitis	197	0	
M124800	Contact dermatitis due to rubber	183	0	
7G0A000	Punch biopsy of lesion of skin of head or neck	181	1	
8Hm0.00	Referral to dermatology clinical assessment service	175	0	
M127800	Photocontact dermatitis [berloque dermatitis]	173	0	
AD30.12	Sarcoptic itch	155	0	
M128300	Allergic contact dermatitis due to dyes	143	0	
M128400	Allergic contact dermatitis due to other chemical products	142	0	
8HJD.00	Dermatology self-referral	135	0	
M12y011	Lanolin contact dermatitis	132	0	
M161G00	Acrodermatitis continua	126	0	
8HTu.00	Referral to eczema clinic	124	1	1
14F1.00	Shave biopsy of lesion of skin NEC	124	1	
M124z00	Contact dermatitis: other chemicals NOS	123	0	
M128100	Allergic contact dermatitis due to cosmetics	121	0	
9NNW.00	Under care of dermatologist	118	0	
M12yA00	Contact dermatitis due to radiation NOS	102	0	
M128000	Allergic contact dermatitis due to adhesives	101	0	
M12yz00	Contact dermatitis: specified agent NOS	99	0	
A544100	Herpes simplex eyelid dermatitis	96	0	
M17y200	Infantile papular acrodermatitis	96	0	
M12y200	Contact dermatitis due to dyes	92	0	
7G0B000	Shave biopsy of lesion of skin of head or neck	90	1	
A532000	Herpes zoster with dermatitis of eyelid	89	0	
M123200	Contact dermatitis due to iodine	74	0	
7G0B300	Shaved deep ellipse biopsy of lesion of skin NEC	74	0	
M21y000	Acrodermatitis atrophicans chronica	73	0	
8HK3.00	Dermatology D.V. requested	68	0	
M12C.00	Radiodermatitis	67	0	
M12y012	Perfume contact dermatitis	61	0	
M128600	Allergic contact dermatitis due to plants, except food	55	0	
8HL3.00	Dermatology D.V. done	53	0	
7G0Ay00	Other specified punch biopsy of skin	53	1	
7G0Cy00	Other specified other biopsy of skin	52	1	

READ Code	Description	Frequency	Possibly Related	Likely Related
M12y300	Contact dermatitis due to furs	49	0	
M12y600	Contact dermatitis due to jewellery	49	0	
13HV400	Seven year itch - marital	49	0	
M124700	Contact dermatitis due to plastic	48	0	
M12yC00	Contact dermatitis due to x-rays	46	0	
AC03.11	Swimmers' itch	44	0	
F4D5.00	Other eyelid infective dermatitis	42	0	
M127z00	Contact dermatitis due to solar radn NOS	42	0	
M126z00	Contact dermatitis due to plants NOS	40	0	
M12y.00	Contact dermatitis due to other specified agents	39	0	
M184.11	Dermatitis factitia	39	0	
M129000	Irritant contact dermatitis due to cosmetics	38	0	
7G0Bz00	Shave biopsy of skin NOS	36	1	
8Hlo.00	Referral to teledermatology service	34	0	
C355000	Acrodermatitis enteropathica	33	0	
M07y200	Dermatitis vegetans	30	0	
M12y100	Contact dermatitis due to cold weather	30	0	
Myu2C00	[X]Other specified dermatitis	29	1	
M145200	Senile dermatitis herpetiformis	27	0	
M128200	Allergic contact dermatitis due drugs in contact with skin	26	0	
M13z.00	Ingestion dermatitis NOS	26	0	
M122100	Contact dermatitis due to cyclohexane	25	0	
M142.00	Juvenile dermatitis herpetiformis	25	0	
SP35200	Serum rash	25	0	
M122z00	Contact dermatitis due to solvent NOS	24	0	
M12y400	Contact dermatitis due to hot weather	21	0	
ZR9..11	DLQI - Dermatology life quality index	21	0	
M118z00	Infantile seborrhoeic dermatitis NOS	20	0	
M121.12	Oil contact dermatitis	20	0	
M126500	Contact dermatitis due to primrose	20	0	
M13y.00	Ingestion dermatitis due to other specified substance	19	0	
M128500	Allergic contact dermatitis due to food in contact with skin	18	0	
M125.00	Contact dermatitis due to food in contact with skin	17	0	
M129400	Irritant contact dermatitis due to plants, except food	17	0	
Myu2300	[X]Allergic contact dermatitis due to other agents	16	0	
M125500	Contact dermatitis due to milk	14	0	
M125z11	Egg contact dermatitis	14	0	
M126100	Contact dermatitis due to poison-ivy	14	0	
M123500	Contact dermatitis due to neomycin	13	0	

READ Code	Description	Frequency	Possibly Related	Likely Related
M124400	Contact dermatitis due to dichromate	13	0	
Myu2000	[X]Other seborrhoeic dermatitis	13	0	
7G0B200	Shaved deep ellipse biopsy of lesion of skin of head or neck	12	0	
7G0By00	Other specified shave biopsy of skin	12	1	
M124300	Contact dermatitis due to caustics	11	0	
M125z00	Contact dermatitis due to food NOS	10	0	
M12C000	Acute radiodermatitis	10	0	
Myu2100	[X]Allergic contact dermatitis due to oth chemical products	10	0	
M124600	Contact dermatitis due to nylon	9	0	
C391211	Thrombocytopenic eczema with immunodeficiency	7	1	0
M126600	Contact dermatitis due to ragweed	7	0	
Myu2700	[X]Unspecified contact dermatitis due to other agents	7	0	
Myu2400	[X]Irritant contact dermatitis due to oth chemical products	6	0	
ZR9..00	Dermatology life quality index	6	0	
M121.11	Grease contact dermatitis	5	0	
M123600	Contact dermatitis due to pediculocides	5	0	
M129100	Irritant contact dermatitis due drugs in contact with skin	5	0	
M12C100	Chronic radiodermatitis	5	0	
Myu2500	[X]Irritant contact dermatitis due to other agents	5	0	
7Q0E.00	High cost dermatology drugs	5	0	
M116.11	Brocq's neurodermatitis	5	1	
F4D4.00	Infective eyelid dermatitis of types resulting in deformity	4	0	
M123100	Contact dermatitis due to fungicides	4	0	
M125200	Contact dermatitis due to flour	4	0	
M124000	Contact dermatitis due to acids	3	0	
M124200	Contact dermatitis due to alkalis	3	0	
M125100	Contact dermatitis due to fish	3	0	
M126300	Contact dermatitis due to poison-sumac	3	0	
M129300	Irritant contact dermatitis due to food in contact with skin	3	0	
M12y700	Contact dermatitis due to light (excluding sunlight)	3	0	
M12y900	Contact dermatitis due to preservatives	3	0	
M15y011	Dermatitis exfoliativa neonatorum	3	0	
M123400	Contact dermatitis due to mercurials	2	0	
M123700	Contact dermatitis due to phenols	2	0	
M125300	Contact dermatitis due to fruit	2	0	
M126000	Contact dermatitis due to lacquer tree	2	0	

READ Code	Description	Frequency	Possibly Related	Likely Related
M12yB00	Contact dermatitis due to ultra-violet rays (excluding sun)	2	0	
M122000	Contact dermatitis due to chlorocompound	1	0	
M122300	Contact dermatitis due to glycol	1	0	
M123000	Contact dermatitis due to arnica	1	0	
M123300	Contact dermatitis due to keratolytics	1	0	
M125000	Contact dermatitis due to cereals	1	0	
M126200	Contact dermatitis due to poison-oak	1	0	
M12y500	Contact dermatitis due to infra-red rays	1	0	
Myu2800	[X]Dermatitis due to other substances taken internally	1	0	
4I82.00	Skin biopsy fibrin level	1	0	
9kM..00	Dermatology management plan given - enhanced services admin	1	0	

Notes: Shading reflects categorization: Black boxes (5) included in algorithm; medium grey likely eczema, light grey possibly eczema

Supplementary Table S2. Treatment Codes

British National Formulary Code	Description
13.00.00.00	Skin
13.01.00.00	Management of skin conditions
13.01.01.00	Vehicles
13.02.00.00	Emollient and barrier preparations
13.02.01.00	Emollient skin preparations
13.02.01.01	Emollient bath additives and shower preparations
13.02.02.00	Barrier preparations
13.03.00.00	Topical local anesthetic and antipruritics
13.04.00.00	Topical corticosteroids
13.05.00.00	Preparations for eczema and psoriasis
13.05.01.00	Preparations for eczema
13.05.03.00	Drugs affecting the immune response
08.01.03.00	Antimetabolites (Methotrexate)
08.02.01.00	Antiproliferative immune suppressants (Azathioprine, Mycophenolate)
08.02.02.00	Other immunosuppresants (Cyclosporin, Tacrolimus)
13.10.01.01	Antibacterial preparations for skin
13.10.01.02	Antibacterial preparations for skin
13.10.03.00	Antiviral preparations for skin
13.05.03.00	Drugs affecting the immune response
14.05.00.00	Interferon gamma
**READ Phototherapy Code**	**Description**
863..11	Phototherapy
8631.00	Phototherapy
8632.11	PUVA/phototherapy
8632.14	UVB phototherapy
8632.15	Ultraviolet light phototherapy
7G0E.00	Phototherapy to skin
7G0E200	Combined photochemotherapy and UVA light therapy to skin
7G0E300	Combined photochemotherapy and UVB light therapy to skin
7G0Ey00	Other specified phototherapy to skin
7G0Ez00	Phototherapy to skin NOS
863..00	Phototherapy/radiation therapy
863Z.00	Radiation/phototherapy NOS
Z6E1.12	Ultraviolet light phototherapy
Z6E1111	UVA phototherapy
Z6E1211	UVB phototherapy
Z6E1411	PUVA phototherapy
Z6E2.00	Photochemotherapy
Z6E5.00	Intermittent phototherapy

Supplementary Table S3. List of exclusionary conditions.*

READ Code	Description
M161000	Psoriasis unspecified
M161z00	Psoriasis NOS
M16..00	Psoriasis and similar disorders
M161600	Guttate psoriasis
M16y000	Scalp psoriasis
M161D00	Pustular psoriasis
M161.00	Other psoriasis
M161F11	Chronic large plaque psoriasis
M161B00	Psoriasis plantaris
M161A00	Psoriasis palmaris
M161F00	Psoriasis vulgaris
M161H00	Erythrodermic psoriasis
M16y.00	Other psoriasis and similar disorders
M161E00	Psoriasis universalis
M161300	Psoriasis diffusa
Myu3000	Other psoriasis
M161C00	Psoriasis punctata
M166.00	Palmoplantar pustular psoriasis
AD30.00	Scabies
AD30.11	Norwegian scabies
Myu2100	Allergic contact dermatitis due to oth chemical products
Myu2300	Allergic contact dermatitis due to other agents
Myu2400	Irritant contact dermatitis due to oth chemical products
Myu2500	Irritant contact dermatitis due to other agents
Myu4300	Other specified erythematous conditions
Myu2700	Unspecified contact dermatitis due to other agents
M128.00	Allergic contact dermatitis
M128200	Allergic contact dermatitis due drugs in contact with skin
M128000	Allergic contact dermatitis due to adhesives
M128100	Allergic contact dermatitis due to cosmetics
M128300	Allergic contact dermatitis due to dyes
M128500	Allergic contact dermatitis due to food in contact with skin
M128400	Allergic contact dermatitis due to other chemical products
M128600	Allergic contact dermatitis due to plants, except food
M12..11	Contact dermatitis
M124000	Contact dermatitis due to acids
M124100	Contact dermatitis due to adhesive plaster

READ Code	Description
M123000	Contact dermatitis due to arnica
M124300	Contact dermatitis due to caustics
M125000	Contact dermatitis due to cereals
M122000	Contact dermatitis due to chlorocompound
M12y100	Contact dermatitis due to cold weather
M122100	Contact dermatitis due to cyclohexane
M120.00	Contact dermatitis due to detergents
M124400	Contact dermatitis due to dichromate
M12y200	Contact dermatitis due to dyes
M125100	Contact dermatitis due to fish
M125200	Contact dermatitis due to flour
M125z00	Contact dermatitis due to food NOS
M125300	Contact dermatitis due to fruit
M123100	Contact dermatitis due to fungicides
M12y300	Contact dermatitis due to furs
M122300	Contact dermatitis due to glycol
M12y400	Contact dermatitis due to hot weather
M12y500	Contact dermatitis due to infra-red rays
M123200	Contact dermatitis due to iodine
M12y600	Contact dermatitis due to jewelry
M123300	Contact dermatitis due to keratolytics
M126000	Contact dermatitis due to lacquer tree
M12y700	Contact dermatitis due to light (excluding sunlight)
M123z00	Contact dermatitis due to medicament NOS
M123400	Contact dermatitis due to mercurials
M12y800	Contact dermatitis due to metals
M125500	Contact dermatitis due to milk
M123500	Contact dermatitis due to neomycin
M124600	Contact dermatitis due to nylon
M124.00	Contact dermatitis due to other chemical products
M123600	Contact dermatitis due to pediculocides
M123700	Contact dermatitis due to phenols
M126.00	Contact dermatitis due to plants
M126z00	Contact dermatitis due to plants NOS
M124700	Contact dermatitis due to plastic
M126100	Contact dermatitis due to poison-ivy
M126200	Contact dermatitis due to poison-oak
M126300	Contact dermatitis due to poison-sumac
M12y900	Contact dermatitis due to preservatives
M126500	Contact dermatitis due to primrose

READ Code	Description
M12yA00	Contact dermatitis due to radiation NOS
M126600	Contact dermatitis due to ragweed
M123800	Contact dermatitis due to scabicides
M127.00	Contact dermatitis due to solar radiation
M127z00	Contact dermatitis due to solar radn NOS
M122z00	Contact dermatitis due to solvent NOS
M12yB00	Contact dermatitis due to ultra-violet rays (excluding sun)
M12yC00	Contact dermatitis due to x-rays
M12z.00	Contact dermatitis NOS
M12zz00	Contact dermatitis NOS
M124z00	Contact dermatitis: other chemicals NOS
M12yz00	Contact dermatitis: specified agent NOS
M125z11	Egg contact dermatitis
M124111	Elastoplast contact dermatitis
M121.11	Grease contact dermatitis
M129.00	Irritant contact dermatitis
M129100	Irritant contact dermatitis due drugs in contact with skin
M129000	Irritant contact dermatitis due to cosmetics
M129300	Irritant contact dermatitis due to food in contact with skin
M129400	Irritant contact dermatitis due to plants, except food
M12y011	Lanolin contact dermatitis
M121.12	Oil contact dermatitis
M12y012	Perfume contact dermatitis
M127000	Unspecified contact dermatitis due to solar radiation
M12y000	Contact dermatitis due to cosmetics
M121.00	Contact dermatitis due to oils and greases
M123.00	Contact dermatitis due to drugs and medicaments
M122.00	Contact dermatitis due to solvents
M129200	Irritant contact dermatitis due to other chemical products
M124800	Contact dermatitis due to rubber
M127800	Photocontact dermatitis [berloque dermatitis]
M101.00	Seborrhoeic dermatitis
M101.11	Seborrhoeic dermatitis capitis
M244.11	Seborrhoea capitis
M263.00	Seborrhoea
M118.00	Infantile seborrhoeic dermatitis
M118000	Infantile seborrhoeic dermatitis capitis
M263100	Seborrhoea faciei
M263z00	Seborrhoea NOS

READ Code	Description
M263000	Seborrhoea corporis
M118z00	Infantile seborrhoeic dermatitis NOS
Myu2000	Other seborrhoeic dermatitis
M263200	Seborrhoea nasi
M263300	Seborrhoea oleosa
M211.12	Ichthyosis - acquired
PH1..00	Ichthyosis congenita
PH14.00	Ichthyosis vulgaris
M211600	Acquired ichthyosis
PH10.00	Congenital ichthyosis, unspecified
PH1z.00	Ichthyosis congenita NOS
PH15.00	X-linked ichthyosis
PH1z.11	Congenital ichthyosiform erythroderma
PH1y.00	Other specified ichthyosis congenita
M127400	Photosensitiveness
M127300	Photodermatitis
M127800	Photocontact dermatitis [berloque dermatitis]
M12A100	Drug photoallergic response
M12A000	Drug phototoxic response
M127900	Hydroa vacciniforme
Q47yy11	Erythroderma neonatorum
PH12.00	Ichthyosiform erythroderma
M130200	Drug-induced erythroderma

Notes: *Based on Eichenfield LF, Tom WL, Chamlin SL, et al. Guidelines of care for the management of atopic dermatitis: section 1. Diagnosis and assessment of atopic dermatitis. *J Am Acad Dermatol.* 2014;70(2):338-351

Supplementary Table S4. Age at diagnosis and last disease activity requiring contact with the physician stratified by children and adults

	Distribution of estimates by source		Difference between physician estimate (from survey) and database	
	Mean	95% CI	Mean	95% CI
**Children**				
Age in years at diagnosis (N=84)				
Physician survey	2.5	(1.9,3.1)	N/A	N/A
Database				
First diagnosis code*	1.9	(1.4,2.4)	0.6	(0.2,1.0)
First prescription for any eczema treatment**	1.6	(1.2, 2.0)	0.8	(0.4, 1.3)
If no symptoms in the year prior to the last visit date, age at last disease activity (N=29)				
Physician survey	5.4	(4.2, 6.8)	N/A	N/A
Database				
First diagnosis code*	5.6	(4.2,6.9)	-0.1	(-1.2,1.1)
First prescription for any eczema treatment**	7.5	(6.2,8.9)	-2.1	(-3.6,-0.5)
**Adults**				
Age in years at diagnosis (N=76)				
Physician survey	34.8	(29.5,40.2)	N/A	N/A
Database				
First diagnosis code*	33.8	(28.6,39.1)	1.0	(-1.3,3.3)
First prescription for any eczema treatment**	34.9	(29.8, 40.0)	-0.1	(-2.7, 2.6)
If no symptoms in the year prior to the last visit date, age at last disease activity (N=24)				
Physician survey	39.1	(28,9, 49.4)	N/A	N/A
Database				
First diagnosis code*	42.0	(32.8,51.2)	-2.8	(-4.9,-0.6)
First prescription for any eczema treatment**	45.2	(35.6,54.7)	-6.0	(-8.5,-3.5)

Notes: *Any of the 5 most commonly used codes (Atopic Dermatitis/ Eczema M111.00, Infantile Eczema M112.00, Flexural Eczema M113.00, Allergic/Intrinsic Eczema M114.00, Eczema NOS M12z100). **See Supplemental Table 2.

Supplementary Table S5. Results of comparison of survey results to UK Working Party Criteria Questions

	N (%) with physician-confirmed eczema that said yes	N (%) with physician-confirmed eczema that said no	Unknown/ missing (% among those who returned a survey)
Has the patient had an itchy skin condition?	144 (88%)	8 (5%)	11 (7%)
Plus, at least 3 of the following:	52 (32%)	1 (4%)	1 (4%)
Does the patient have a history of generally dry skin?	64 (39%)	38 (23%)	61 (37%)
Has the patient ever had *visible* flexural dermatitis?	75 (46%)	49 30%)	39 (24%)
Does the patient have a *history of* flexural dermatitis?	56 (34%)	66 (40%)	41 (25%)
Does the patient have a history of other atopic disease?*	56 (34%)	81 (50%)	26 (16%)
Onset under age 2?	65 (40%)	95 (58%)	3 (1%)

Notes: *e.g. asthma or allergic rhinitis or in a first degree relative if under age 4

Supplemental Figure S1. Bland Altman Plots of difference between survey and database estimates against mean

Figure 1a. Survey diagnosis age vs database diagnosis age based on first diagnosis code
Limits of agreement (Reference Range for difference): -13.107 to 14.706
Mean difference: 0.800 (CI -0.286 to 1.885)*
Range : 0.055 to 75.167
Pitman's Test of difference in variance: r = 0.056, n = 160, p = 0.478

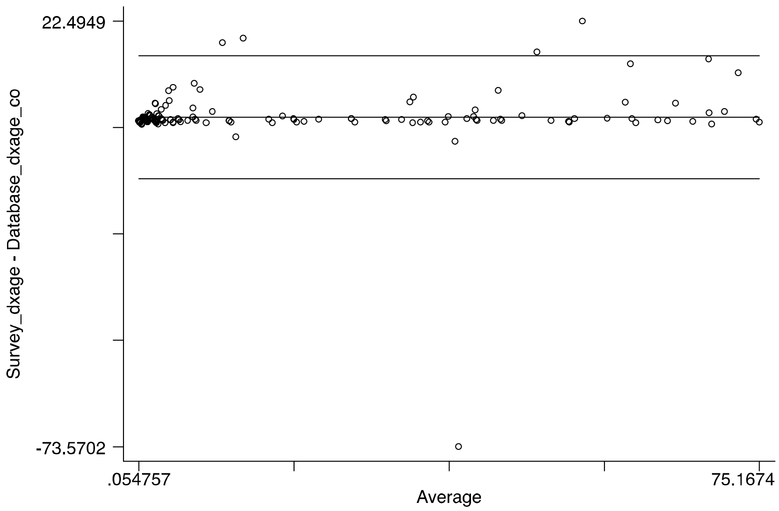

Figure 1b. Survey diagnosis age vs database diagnosis age based on first prescription code
Limits of agreement (Reference Range for difference): -15.714 to 16.542
Mean difference: 0.414 (CI -0.845 to 1.673)*
Range : -0.171 to 75.178
Pitman's Test of difference in variance: r = 0.023, n = 160, p = 0.775

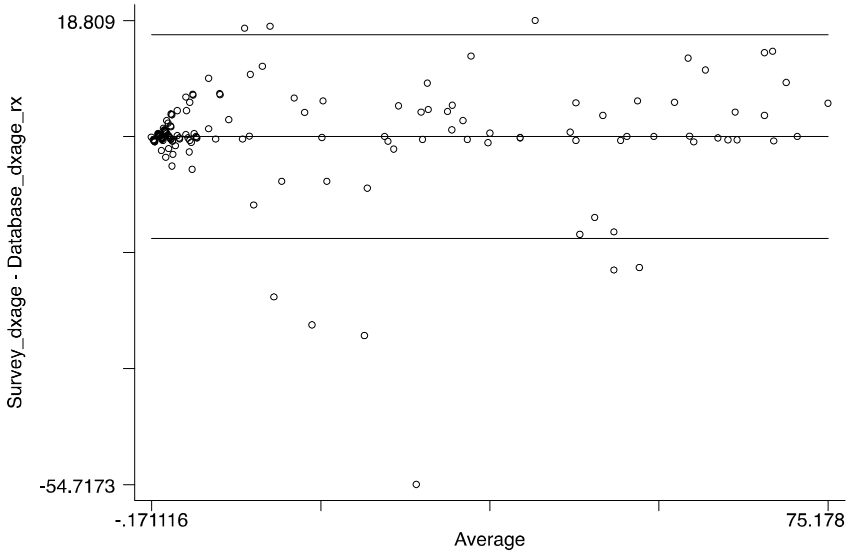

Figure 1c. Survey age at last disease activity vs database age at last disease activity based on last diagnosis code

Limits of agreement (Reference Range for difference): -9.899 to 7.285
Mean difference: -1.307 (CI -2.491 to -0.123)*
Range : 0.703 to 80.350
Pitman's Test of difference in variance: r = 0.004, n = 53, p = 0.976

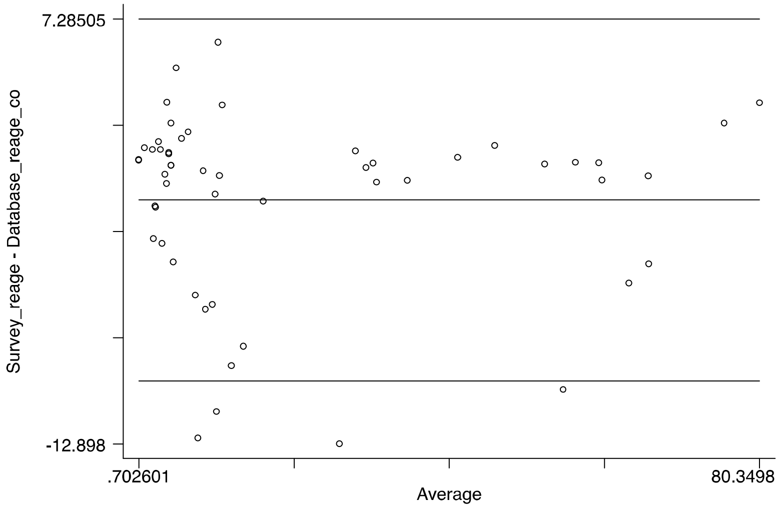

Figure 1d. Survey age at last disease activity vs database age at last disease activity based on last prescription code

Limits of agreement (Reference Range for difference): -14.515 to 6.818
Mean difference: -3.849 (CI -5.319 to -2.379)*
Range : 0.985 to 81.030
Pitman's Test of difference in variance: r = -0.149, n = 53, p = 0.286

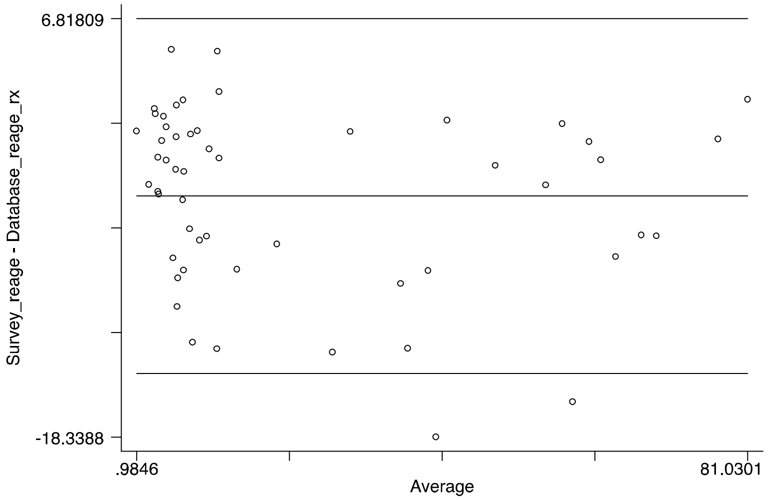

Notes: *The difference between the database and survey estimates did not follow a normal (Gaussian) distribution; therefore the 95% of the differences may not lie within 2 standard deviations of the mean.

Supplementary Figure S2. Calculation of positive predictive value (PPV)

Codes for eczema	Confirmation of eczema by physician survey: Yes	Confirmation of eczema by physician survey: No
Yes	A	B
No	C	D

PPV = A / (A+B)

Supplementary Figure S3. Survey Instrument (Adults)

Questionnaire

Practice ID	Patient ID	Year of Birth	Sex

Please complete this survey to help us validate the diagnosis of **ECZEMA*** in routine health records.
*Eczema is also known as atopic dermatitis or atopic eczema.

Has the patient had an itchy skin condition?
[] YES [] NO [] UNKNOWN

Does the patient have a history of generally dry skin?
[] YES [] NO [] UNKNOWN

Has the patient ever had *visible* flexural dermatitis involving the skin creases, such as the fronts of elbows, behind the knees, fronts of ankles, around the neck, or around the eyes?
[] YES [] NO [] UNKNOWN

Does the patient have a *history of* flexural dermatitis?
[] YES [] NO [] UNKNOWN

Does the patient have a history of other atopic disease (e.g asthma or allergic rhinitis)?
[] YES [] NO [] UNKNOWN

Based on your knowledge of this patient and review of the medical record, has the patient ever had eczema?
[] YES [] NO *(End of survey, thank you)!*

↓

Did the patient's eczema begin at approximately the following date? MM-YYYY
[] YES [] NO - *If no*, at approximately what age did the patient's eczema begin?
____ years [] UNKNOWN

When was the last time the patient was seen prior to 1/1/2013? ____-______ (MM-YYYY)
Did the patient have eczema symptoms during the year prior to that visit?
[] YES [] NO [] UNKNOWN

If yes, please provide a global assessment of the **severity** of the patient's eczema during the year prior to that visit:
[] Mild: areas of dry skin plus infrequent itching (with or without small areas of redness)
[] Moderate: areas of dry skin plus frequent itching plus redness (with or without excoriation and localised skin thickening)
[] Severe: widespread areas of dry skin plus incessant itching plus redness (with or without excoriation, extensive skin thickening, bleeding, oozing, cracking and alteration of pigmentation)

If no, please specify the patient's approximate age at eczema **remission:**
[] ____years
[] UNKNOWN

End of survey, thank you!

[illegible]

Supplementary Figure S4. Survey Instrument (Children)

Questionnaire

Practice ID	Patient ID	Year of Birth	Sex

Please complete this survey to help us validate the diagnosis of ECZEMA* in routine health records.
*Eczema is also known as atopic dermatitis or atopic eczema.

Has the patient had an itchy skin condition?
[ ] YES [ ] NO [ ] UNKNOWN

Does the patient have a history of generally dry skin?
[ ] YES [ ] NO [ ] UNKNOWN

Has the patient ever had *visible* flexural dermatitis involving the skin creases, such as the fronts of elbows, behind the knees, fronts of ankles, around the neck, or around the eyes (or visible dermatitis on the cheeks and/or extensor areas in children aged 18 months or under)?
[ ] YES [ ] NO [ ] UNKNOWN

Does the patient have a *history* of flexural dermatitis (or dermatitis on the cheeks and/or extensor areas if aged 18 months or under)?
[ ] YES [ ] NO [ ] UNKNOWN

Does the patient have a history of other atopic disease (e.g. asthma or allergic rhinitis), **OR** a family history of atopic disease in a first degree relative if aged under 4 years?
[ ] YES [ ] NO [ ] UNKNOWN

Based on your knowledge of this patient and review of the medical record, has the patient ever had eczema*?
[ ] YES [ ] NO *(End of survey, thank you)!*

Did the patient's eczema begin at approximately the following date? MM-YYYY
[ ] YES [ ] NO - *If no*, at approximately what age did the patient's eczema begin?
____ years [ ] UNKNOWN

When was the last time the patient was seen prior to 1/1/2013? ____-______ (MM-YYYY)
Did the patient have eczema symptoms during the year prior to that visit?
[ ] YES [ ] NO [ ] UNKNOWN

If yes, please provide a global assessment of the **severity** of the patient's eczema during the year prior to that visit:
[ ] Mild: areas of dry skin plus infrequent itching (with or without small areas of redness)
[ ] Moderate: areas of dry skin plus frequent itching plus redness (with or without excoriation and localised skin thickening)
[ ] Severe: widespread areas of dry skin plus incessant itching plus redness (with or without excoriation, extensive skin thickening, bleeding, oozing, cracking and alteration of pigmentation)

If no, please specify the patient's approximate age at eczema **remission:**
[ ] ____years
[ ] UNKNOWN

End of survey, thank you!
